# The emerging specialty of perioperative medicine: a UK survey of the attitudes and behaviours of anaesthetists

**DOI:** 10.1186/s13741-019-0132-0

**Published:** 2020-01-21

**Authors:** J. S. L. Partridge, A. Rogerson, A. L. Joughin, D. Walker, J. Simon, M. Swart, J. K. Dhesi

**Affiliations:** 1 Perioperative Medicine for Older People Undergoing Surgery (POPS), Older Persons Assessment Unit, Guy’s and St Thomas’ NHS Foundation Trust, Guy’s Hospital, Great Maze Pond, London, SE1 9RT UK; 2 Division of Primary Care and Public Health Sciences, Faculty of Life Sciences and Medicine, King’s College London, London, UK; 3 Perioperative Medicine for Older People Undergoing Surgery (POPS), Guy’s and St Thomas’ NHS Foundation Trust, London, UK; 4 Department of Medicine for the Elderly, Great Western Hospital, Marlborough Road, Swindon, UK; 5 Anaesthesia and Critical Care Medicine, University College London Hospitals, London, UK; 6 Imperial College Healthcare NHS Trust, London, UK; 7 Anaesthesia and Perioperative Medicine, Torbay Hospital, Torquay, Devon TQ2 7AA UK; 8 National Clinical Lead for the Perioperative Medicine Programme, Royal College of Anaesthetists, London, UK; 9 Division of Primary Care and Public Health Sciences, Faculty of Life Sciences and Medicine, King’s College London, London, UK

**Keywords:** Perioperative medicine, Barriers and facilitators, Service development, Education and training

## Abstract

**Background:**

In 2014, the Royal College of Anaesthetists (RCoA) launched the Perioperative Medicine Programme to facilitate the delivery of best preoperative, intraoperative and postoperative care through implementation of evidence-based medicine to reduce variation and improve postoperative outcomes. However, variation exists in the establishment of perioperative medicine services in the UK. This survey explored attitudes and behaviours of anaesthetists towards perioperative medicine, described current anaesthetic-led perioperative medicine services across the UK and explored barriers to anaesthetic involvement in perioperative medicine.

**Methods:**

Survey content based on the RCoA vision document was refined and validated using an expert panel. An anonymous electronic survey was then sent by email to the members of the RCoA.

**Results:**

Seven hundred fifty-eight UK anaesthetists (4.5% of the RCoA mailing list) responded to the survey. Of these, 64% considered themselves a perioperative doctor, with 65% having changed local services in response to the RCoA vision. Barriers to developing perioperative medicine included insufficient time (75%) and inadequate training (51%). Three quarters of respondents advocate anaesthetists leading the development of perioperative medicine.

**Conclusions:**

Despite evidence of emerging services, this survey describes barriers to ongoing development of perioperative medicine. Facilitators may include increased clinical exposure, targeted education and training and collaborative working with other specialties.

## Background

The number of surgical procedures performed annually in the NHS is approximately 5.1 million and rising (Abbott et al., 2017). A high-risk subgroup (12.5–20%) of surgical patients in the UK (Pearse et al., 2006; Fowler et al., 2019; Findlay et al., 2001) accounts for a significant proportion of postoperative hospital mortality (Pearse et al., 2006; Fowler et al., 2019) attributed in part to medical complications (Findlay et al., 2001; Royal College of Anaesthetists Perioperative Medicine, 2015; Patel & Zenilman, 2001), and resulting in significant increased financial cost. In response, the Royal College of Anaesthetists (RCoA) launched the Perioperative Medicine Programme in 2014 (Royal College of Anaesthetists Perioperative Medicine, 2015). This initiative aims to promote the best preoperative, intraoperative and postoperative care through evidence-based medicine to reduce variation and improve postoperative outcomes. Similar programmes have also been established in other settings, such as the Perioperative Surgical Home in Australia and the United States (Perioperative Surgical Home Australia, 2019; American Society of Anaesthesiologists, 2019).

To support the implementation of this programme, a number of actions have been undertaken in the UK. Hospitals have appointed perioperative medicine leads and begun to reorganise anaesthetic departments to develop the specialty of perioperative medicine, underpinned by the Guidelines for the Provision of Anaesthetic Services (GPAS) documents (Royal College of Anaesthetists, 2018). The anaesthetic higher specialist curriculum (Royal College of Anaesthetists, 2010) has been revised to include competencies in perioperative medicine and formalised training opportunities including postgraduate qualifications now exist. To raise standards and reduce variation, national quality improvement projects and research such as the Perioperative Quality Improvement Programme (PQIP) (Perioperative Quality Improvement Program, 2019) have been established with real-time results informing iterative changes in clinical services.

Despite these changes, a recent survey of perioperative leads (Perioperative Quality Improvement Program, 2019) demonstrated wide variation in the delivery of perioperative medicine services by anaesthetists in routine clinical practice. Understanding the barriers to implementing perioperative medicine from the perspective of anaesthetists as opposed to perioperative medicine leads may facilitate the further development of this specialty. This national UK survey, performed jointly by the RCoA and the British Geriatrics Society’s Perioperative Medicine for Older People (POPS) Specialist Interest Group aims to:

- Explore the attitudes and behaviours of anaesthetists towards perioperative medicine
- Describe current anaesthetist-led perioperative medicine services across the UK
- Explore barriers to anaesthetists’ involvement in perioperative medicine

## Methods

Ethical approval was not sought because participation was voluntary and patients were not involved.

The survey content was based on the RCoA perioperative medicine vision document. An expert panel developed 21 multiple choice, ranking and Likert questions. The survey was reviewed for readability, non-ambiguity and content validity by a separate expert panel achieving a score of 0.63 (Lawshe, 1975) which is above the validity threshold of 0.62 for ten expert raters. The validated survey was piloted by a convenience sample of consultant and trainee anaesthetists.

The survey was sent electronically using Survey Monkey software to the RCoA fellowship to maximise responses. The survey specifically invited all UK anaesthetic consultants and trainees above ST5 to respond. The invitation email included information about the study and the web link to participate. Reminders were sent on three occasions over a 4-month period. RCoA endorsement was used a further measure to improve response rate.

Responses were analysed using descriptive statistics and reported by themes. In some questions, respondents were invited to mark all options which applied. For this reason, the results may add up to over 100% in some questions.

## Results

Seven hundred fifty-eight anaesthetists responded, 61% of whom were consultants and the remainder trainees or specialty and associate specialist (SAS) doctors. The fellowship email list held by the RCoA contains 16,744 email addresses giving a theoretical response rate of 4.5%. Sixty-five percent of respondents worked at a teaching hospital, 36% at a district general hospital and 5% from private or other institution types. Fifty-three percent confirmed that there is a perioperative medicine lead at the trust where they work, and 4% of respondents were perioperative medicine leads. The remainder stated that there was no lead (22%) or did not know if there was a perioperative lead in position (25%).

### Attitudes to perioperative medicine

Sixty-four percent of respondents consider themselves to be a perioperative doctor. Forty-three percent had watched the RCoA video on the vision for perioperative care, and 25% had instigated changes within their hospital based on RCoA recommendations.

Differences existed in when anaesthetists believe the perioperative pathway begins, with the majority of respondents stating it begins in either primary care (68%) or the surgical outpatient clinic (49%). The predominant view was that anaesthetists should be involved in the perioperative pathway from the time of decision to operate (54%). Forty-four percent of respondents felt anaesthetists should be involved in the perioperative pathway until there were no acute postoperative clinical issues, rather than until discharge from hospital. The key components of perioperative care, as described by the respondents, are summarised in Table 1.

**Table 1:** The views of respondents regarding the key components of perioperative care.

More commonly considered a key component of perioperative care (> 50% responded)	Less commonly considered a key component of perioperative care (< 50% responded)
Preoperative assessment (96%)	Rehabilitation goal setting/discharge planning (48%)
Identification of comorbidity (93%)	Longer term post-discharge follow-up of medical complications (37%)
Medication optimisation (93%)	Taking consent for surgery (33%)
Anaesthetic planning (93%)	Following up surgical complications (24%)
Risk quantification (92%)	
Anaesthetising the patient (88%)	
Identification of geriatric syndromes (81%)	
Consent for anaesthesia (79%)	
Managing postoperative medical complications (79%)	
Assessment of capacity to consent to procedure (66%)	
Postoperative pharmacological review (57%)	

Although these were considered important components of perioperative medicine, the free text comments suggested that respondents felt that the delivery of all components is outside the remit of the anaesthetist.

### Preoperative assessment

**Fig. 1:**
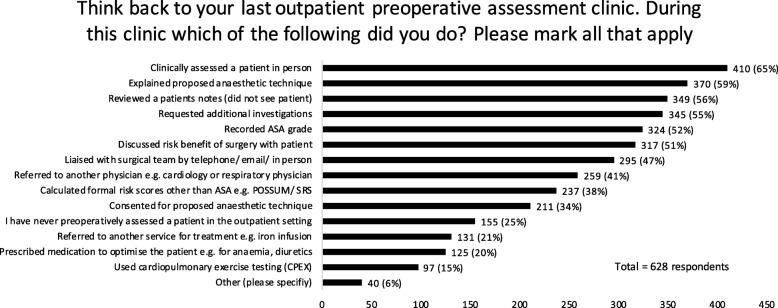
The nature of preoperative assessment reviews and interventions

Eighty-two percent of respondents confirmed that there is a named clinical lead for preoperative assessment at their trust. Forty percent of consultant respondents had dedicated preoperative assessment outpatient sessions. The nature of preoperative assessment and the preoperative interventions delivered by respondents (*n* = 628) are summarised in Fig. 1.

Fifty-four percent of anaesthetists had contact with preoperative assessment nurses on a weekly or monthly basis despite only 14% of respondents having dedicated sessions in their job plan to support preoperative assessment nurses. Forty-six percent of respondents said that their last such contact was face-to-face.

Sixty-seven percent confirmed that cardiopulmonary exercise testing (CPET) was available in the centre where they work, for the preoperative assessment of elective surgical patients. Anaesthetists were most often the clinician responsible for interpreting CPET results. The most frequently cited uses of CPET (specifically the last CPET result seen) were risk stratification (*n* = 354, 63%), postoperative resource planning (*n* = 311, 55%) and shared decision-making (*n* = 227, 40%). When reflecting on the last preoperative clinic patient they reviewed, 15% of respondents stated that CPET results did not change their management, whilst five percent reported that it prompted cancellation of surgery. Of the 100 free text comments, 85 stated that they never performed CPET or cannot remember the last time they had seen a patient with a CPET result.

### Postoperative care

**Fig. 2:**
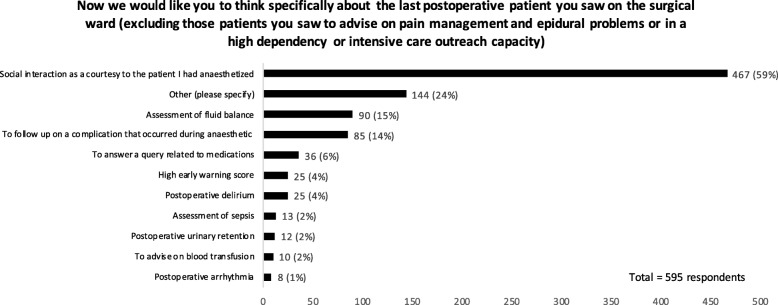
The nature of postoperative ward reviews

The nature of respondents’ postoperative reviews is shown in Fig. 2. Thirty percent of respondents had not performed postoperative reviews in the past week. Of those who did attend a patient postoperatively on the ward, ‘social interaction as a courtesy to the patient’ was the most commonly cited indication for the review (*n* = 467, 79%). The remaining options (assessment of fluid balance, postoperative delirium, high early warning score, postoperative arrhythmia, to follow-up on a complication that occurred during anaesthetic (e.g. allergy, hypotension), postoperative urinary retention, to answer a query related to medications, assessment of sepsis, to advise on blood transfusion) were selected by 15% respondents or less. Analgesia review was frequently mentioned in free text comments.

**Fig. 3:**
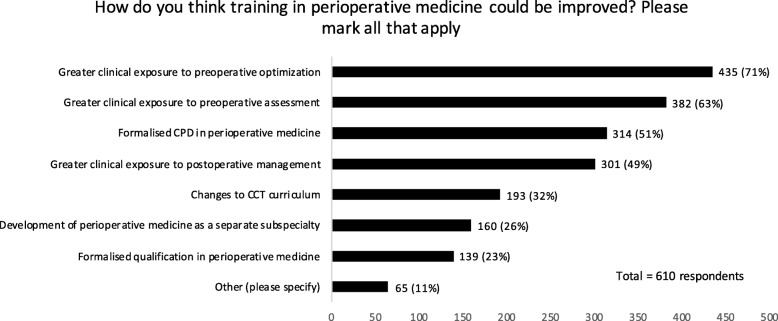
Anaesthetists’ views on improving perioperative medicine training

### Attitudes to the specialty of perioperative medicine

There was a spread of opinion on the statement *The CCT in Anaesthetics’ curriculum gives adequate emphasis to perioperative medicine, including preoperative assessment, preoperative optimisation and postoperative medical management*. Thirty-nine percent agreed or strongly agreed, 27% disagreed or strongly disagreed and 33% neither agreed nor disagreed. When asked if respondents were confident in the current practice of optimising medical comorbidity, only 26% agreed or strongly agreed (27% neither agreed nor disagreed, 47% disagreed/strongly disagreed).

Anaesthetists’ views on how perioperative medicine training can be improved are described in Fig. 3. The majority of respondents advocate increased clinical exposure in pre- and postoperative settings (total 610 respondents).

Specifically, over half of respondents felt that they would value training through greater clinical exposure to postoperative management.

Seventy-eight percent agreed or strongly agreed that anaesthetists are uniquely placed to lead the future development of perioperative medicine. Questioned on which specialties should deliver perioperative medicine, respondents cited anaesthetists most frequently (91%), followed by geriatricians (67%). The spread of respondents advocating that surgeons, intensivists and general physicians deliver perioperative medicine was similar at 53–59% of respondents, with just 33% respondents suggesting that organ-specific physicians should deliver care.

### Barriers to anaesthetic input into perioperative medicine

When asked about barriers to anaesthetists’ involvement in perioperative medicine, 76% of respondents cited insufficient room in current job plans, and 66% reported that there are too few anaesthetists to deliver perioperative medicine. Forty-two percent described a high financial cost to implementing perioperative services and 31% believed that such initiatives would not be supported by management. Furthermore, 51% reported that training in perioperative medicine for anaesthetists is inadequate, with 41% stating that anaesthetists do not want to practice perioperative medicine.

Just over half of respondents (51%) disagreed with the statement ‘physicians are better placed to deliver perioperative medicine’ with just 18% agreeing or strongly agreeing. Twelve percent of respondents stated that ‘physicians are encroaching on perioperative medicine’. Full details on perceived barriers to anaesthetic input into perioperative medicine are given in Table 2.

**Table 2:** Perceived barriers to anaesthetic input into perioperative medicine

	Strongly agree	Agree	Neither	Disagree	Strongly disagree
Anaesthetists do not want to practice perioperative medicine	9%	31%	24%	30%	6%
	53	197	151	182	35
Increased anaesthetic input into the perioperative pathway will not affect patient outcomes	1%	5%	14%	59%	21%
	9	28	87	368	127
There is no room in current job plans	35%	41%	17%	6%	1%
	214	251	102	42	7
Anaesthetists lack sufficient training in perioperative medicine	8%	43%	22%	22%	5%
	46	266	138	138	28
Physicians are better placed to deliver perioperative medicine	3%	14%	31%	38%	14%
	22	87	189	234	82
Surgical teams are reluctant to allow increased anaesthetic input into perioperative services	2%	16%	24%	46%	12%
	15	97	151	280	75
The implementation of innovative pathways of care is not supported by the management at my trust	7%	24%	39%	27%	3%
	44	147	238	164	21
The cost of increasing perioperative services would be too high	8%	35%	34%	19%	4%
	50	213	210	120	23
There are not enough anaesthetists to deliver perioperative medicine	27%	48%	11%	12%	2%
	165	300	65	75	10
There is a lack of evidence linking increased perioperative anaesthetic input with improved patient outcomes	3%	21%	38%	32%	6%
	19	131	230	199	38
Physicians are encroaching on perioperative medicine	1%	10%	30%	50%	9%
	9	63	182	303	57

### Local initiatives

Respondents were invited to leave free text answers describing changes implemented at the hospital where they work in response to recommendations in the RCoA perioperative vision document. Responses cited three or more times were included. Local clinical initiatives include the appointment of a trust lead in perioperative medicine and anaesthetic consultant involvement in preoperative assessment and specialty-specific preoperative clinics. Joint anaesthetic, surgical and geriatric medicine clinics have been established with expansion of postoperative high-dependency units. Preoperative risk assessment processes have been refined through CPEX testing and embedding routine frailty scoring. Pathway development has included anaemia and intravenous iron infusion services, expansion of enhanced recovery after surgery (ERAS) protocols and establishment of postoperative liaison pathways. In terms of education and training, respondents describe increasing trust perioperative medicine teaching through training modules, creation of fellowships and quality improvement work.

## Discussion

This is the first study to report attitudes and behaviours of UK anaesthetists regarding perioperative medicine. Building on a survey of perioperative medicine leads (Bougeard et al., 2017) and early adopters or enthusiasts for the RCoA perioperative medicine vision (Groves et al., 2017), this work aimed to examine the views of all anaesthetists regarding perioperative medicine. Although only 40% of respondents had seen the RCoA Perioperative Medicine film in the 3 years since this vision was articulated, a quarter of anaesthetists describe resultant changes in the provision of local clinical services with about half of respondents reporting an awareness of their local perioperative medicine lead.

On the whole there is concordance between the specialty and the RCoA in the definition of the perioperative pathway. However, whilst the college advocates anaesthetic involvement from the point of contemplation for surgery, respondents to this survey report their wish to be involved later in the pathway once the decision to operate has been made. This subtle distinction may reflect the difference between shared decision-making, which involves a ‘whether to operate’ discussion, and a focus on the ‘how to anaesthetise’ discussion once the decision to operate has already been made.

At present, preoperative services are frequently delivered by preoperative assessment clinic nurses supported by anaesthetists. This survey provides further detail on routine clinical practice. Only half of the respondents describe regular contact with preoperative assessment nurses and the majority reported seeing the patients in person and explaining the proposed anaesthetic technique. Despite this, a large number of respondents described assessing patients through notes review and only 14% of respondents are allocated time in their job plan for preoperative assessment. The utility of notes review is unclear with considerable resource implications in using a consultant workforce for this activity. Additionally, in half of cases, respondents described onward referral to physicians for medical assessment and optimisation and this may reflect the finding that three quarters of respondents did not feel confident in current evidence-based preoperative medical optimisation. In addition, the reported lack of training in managing multimorbidity may also impact on scarce resource through multiple onward referrals. This has implications for job planning, workforce projections and training.

Anaesthetists clearly describe postoperative management of the patient being within the remit of the perioperative physician. The majority of anaesthetists do attend the patient postoperatively; however, in most cases, this is conducted as a courtesy or to review postoperative analgesia requirements, rather than for postoperative medical management. This may reflect the reported need for further training in the postoperative phase of perioperative medicine which is increasingly being realised as a key stage in minimising functional deterioration, promoting timely discharge and achieving quality as defined by patient reported outcomes. Alternatively, this finding may represent a discrepancy between what anaesthetists state the role of a perioperative physician is and what occurs in practice whether due to behaviours or job planning restrictions. The GPAS document may help to define the role of a perioperative physician and may be useful for clinical directors to produce accurate job descriptions for the expansion of perioperative positions. Such clarification may facilitate ‘buy in’ from medical directors and commissioners. Although the RCoA vision has been successful in rebranding perioperative medicine, there is a need for a comprehensive reorganisation of services and clarity regarding clinical roles in order to meaningfully translate the vision into clinical practice.

Three quarters of anaesthetists state that they are uniquely placed to lead the future development of perioperative medicine. Concurrently, a paradoxic but key finding is that forty percent of respondents did not want to practice perioperative medicine. This disconnect requires further exploration and may result in a divergent training option for anaesthetic trainees. Learning from the intensive care training model, a perioperative medicine curriculum could be open to anaesthetists, physicians and surgeons with a focus on the whole patient pathway encompassing preoperative, intraoperative, postoperative training and shared decision-making. Such an approach may address the training issue raised in this survey where anaesthetists reported that, at present, confidently managing all components of perioperative medicine is outside their remit. Similarly with two-thirds of respondents reporting a perceived role for geriatricians, the scope for collaborative working is apparent. These issues are relevant in light of the GMC Greenaway report circa 2013, which called for a modernisation of medical training to meet the demands of the changing patient population (Kumar et al., 2013; Shape of Training, 2013). In response to this new models of training are emerging with a focus on equipping junior doctors with knowledge and skills in ‘whole patient’ and ‘whole pathway’ perioperative medicine (Rogerson et al., 2018; Braude et al., 2016).

In addition to these workforce and training barriers, adequate funding remains key to delivering perioperative medicine services. Over a third of respondents cited a lack of time in their job plan as a limitation to developing local perioperative medicine services. This resonates with the findings of a national survey of geriatrician delivered perioperative medicine services which also identified inadequate funding as the predominant barrier to developing services in the UK (Joughin et al., 2019).

Despite a response from 758 anaesthetists, the reported response rate was low at 4.5%. This likely represents an underestimate of the actual response rate as the RCoA mailing list includes doctors of all grades, allied health professionals, overseas RCoA members and outdated email contacts. In addition, the low response rate may reflect a degree of apathy regarding this subject and/or survey fatigue. Steps to address these issues included clear instructions regarding sampling frame in initial email, follow-up emails to encourage response and RCoA endorsement. Furthermore, polarity regarding the issue of perioperative medicine persists within the anaesthetic community (McGlennan et al., 2015) which may have had a bearing on response rate, potentially reflecting a lack of interest in perioperative medicine. Conversely, there may be bias in responses received skewing towards representation of the views of perioperative medicine enthusiasts. In addition, the possibility of large numbers of respondents being from a small number of localities and thus introducing bias cannot be excluded.

## Conclusions

In conclusion, a majority of UK anaesthetists in this survey believe that they are uniquely placed to lead the future development of perioperative medicine, and many trusts are implementing a variety of measures to enhance perioperative care. However, workforce insufficiency and lack of training in the specialty appear to be the main barriers to effecting change. In addition, engaging the two-fifths of anaesthetists who expressed a lack of interest in perioperative medicine remains a focus for the development of the specialty if all anaesthetists are to practice perioperative medicine. Facilitators may include increased clinical exposure, targeted education and training, innovative perioperative medicine training programmes and collaborative working with other specialties.

## Data Availability

The datasets used and/or analysed during the current study are available from the corresponding author on reasonable request.

## Appendix

### Survey Questions

1. Please mark the grade at which you work. Tick one answer only.

- Consultant
- SAS Grade
- Specialist trainee (post FRCA)
- Specialist trainee (pre FRCA)
- Core/ACCS trainee
- Other (please specify)

2. Please mark your current place of work. Please tick all that apply.

- Teaching hospital
- District general hospital
- Private sector hospital
- Other (please specify)

3. Do you consider yourself to be a perioperative doctor?

- Yes
- No
- Not sure

4. At the trust where you work is there a named lead for perioperative medicine?

- Yes
- No
- Not sure

5. Are you the clinical lead for perioperative medicine?

- Yes
- No
- N/A

6. When do you think the perioperative pathway of care (for elective surgery) starts? Please mark all that apply.

- Primary care (once possible need for surgery is identified)
- Surgical clinic (once surgery is offered as a treatment option)
- Preoperative assessment clinic
- On admission to hospital for surgery
- In the anaesthetic room
- Other (please specify)

7. When do you think the anaesthetists’ involvement in the care of surgical patients should start? Please tick one option only.

- When patient is referred to surgical OP clinic
- When the decision to operate is made
- At preoperative assessment clinic
- On the day of admission to surgical unit
- In the anaesthetic room
- Other (please specify)

8. When do you think the anaesthetists’ involvement in the care of surgical patients should end? Please tick one option only.

- In the recovery room
- At a fixed point after surgery (e.g. 24 hours, day 3 etc)
- Once there are no acute clinical issues and the patient is awaiting rehab or community based care
- Once the patient is discharged from hospital
- Other (please specify below)

9. What do you consider to be the key components of perioperative care? Please mark all that apply.

- Preoperative assessment
- Quantification of perioperative risk
- Identification of co-morbidity
- Identification of geriatric syndromes (e.g. frailty or cognitive impairment)
- Medical optimisation (e.g. treatment of anaemia, heart failure)
- Anaesthetic planning
- Assessment of capacity to consent to procedure
- Taking consent for anaesthesia
- Taking consent for surgical procedure
- Anaesthetising the patient
- Management of postoperative medical complications
- Postoperative rehabilitation, goal setting and discharge planning
- Follow up of surgical complications (e.g. reduced range of movement following joint replacement)
- Longer term post discharge follow-up of postoperative medical complications (e.g. delirium, acute kidney injury)
- Postoperative pharmacological review (e.g. recommencement of cardiac medications stopped during the perioperative period)
- Other (please specify)

10. At the hospital where you work do you have a named clinical lead for preoperative assessment?

- Yes
- No
- Not sure

11. Are you the clinical lead for preoperative assessment?

- Yes
- No
- Not sure

12. If you are a consultant, do you have dedicated outpatient sessions to preoperatively assess elective patients?

- Yes
- No
- Not sure
- I am not a consultant

13. Think back to your last outpatient preoperative assessment clinic. During this clinic which of the following did you do? Please mark all that apply.

- Reviewed a patient’s notes (did not see patient)
- Clinically assessed a patient in person
- Liaised with surgical team by telephone / email / in person
- Requested additional investigations
- Recorded ASA grade
- Calculated formal risk scores other than ASA e.g. POSSUM / SRS
- Utilised cardiopulmonary exercise testing (CPEX)
- Prescribed medication to optimise the patient e.g. iron for anaemia, diuretics for heart failure
- Referred to another physician e.g. cardiology or respiratory physician
- Referred to another service for treatment e.g. iron infusion
- Discussed risk benefit of surgery with patient
- Explained proposed anaesthetic technique
- Consented for proposed anaesthetic technique
- I have never preoperatively assessed a patient in the outpatient setting
- Other (please specify)

14. Do you have dedicated sessions in your job plan to liaise with preoperative assessment clinic nurses?

- Yes
- No
- Not sure

15. In my current practice I have contact with preoperative assessment clinic nurses;

- Daily
- Weekly
- Monthly
- Yearly
- Never
- Other (please specify)

16. We would now like you to think about the last clinical contact you had with a preoperative assessment clinic nurse. Was this by;

- Email
- Telephone
- Face to face
- I have never had contact with a preoperative clinic nurse
- Other (please specify)

17. Do any elective surgical patients operated on within your hospital undergo preoperative CPEX testing?

- Yes
- No
- Not sure

18. At the hospital where you work who interprets CPEX results? Mark all that apply.

- Anaesthetists
- Physicians e.g. respiratory
- CPEX technicians
- Don’t know
- Other (please specify)

19. Now think back to the last patient you anaesthetised in whom you had a preoperative CPEX result. What did you use the result for? Please mark all that apply.

- Risk stratification
- To optimise the patient preoperatively, e.g. treat anaemia, alter COPD medications
- To inform the setting for postoperative care e.g. critical care
- To modify the surgical procedure e.g. less invasive procedure
- To alter the proposed anaesthetic technique
- To ensure shared decision making with patient
- To cancel the surgery
- The CPEX result did not alter our management
- Other (please specify)

20. Think back over your last week at work. During the last week how many times did you visit a postoperative patient on a surgical ward (excluding those patients you saw to advise on pain management and epidural problems)?

- I didn’t see any postoperative patients on the ward in the last week
- Once
- 2-4 times
- 5-7 times
- >7 times

21. Now we would like you to think specifically about the last postoperative patient you saw on the surgical ward (excluding those patients you saw to advise on pain management and epidural problems or in a high dependency or intensive care outreach capacity). What was the focus of your consultation? Please mark all those that apply.

- Social interaction as a courtesy to a patient I had anaesthetised
- Assessment of fluid balance
- Postoperative delirium
- High Early Warning Score
- Postoperative arrhythmia
- To follow up on a complication that occurred during anaesthetic (e.g. allergy, hypotension)
- Postoperative urinary retention
- To answer a query related to medications
- Assessment of sepsis
- To advise on blood transfusion
- Other (please specify)

22. Please indicate how strongly you agree with the statement below. ‘The CCT in Anaesthetics’ curriculum gives adequate emphasis to perioperative medicine, including preoperative assessment, preoperative optimisation and post operative medical management'.

- Strongly agree
- Agree
- Neither agree nor disagree
- Disagree
- Strongly disagree

23. Please indicate how strongly you agree with the statement below. ‘I am up to date with current practice in optimisation of medical conditions (e.g. changing heart failure drugs, prescribing antihypertensives etc).’

- Strongly agree
- Agree
- Neither agree nor disagree
- Disagree
- Strongly disagree

24. How do you think training in perioperative medicine could be improved? Please mark all that apply.

- Greater clinical exposure to preoperative assessment
- Greater clinical exposure to preoperative optimisation
- Greater clinical exposure to postoperative management
- Formalised CPD in perioperative medicine
- Formalised qualification in perioperative medicine
- Changes to CCT curriculum
- Development of perioperative medicine as a separate subspecialty
- Other (please specify)

25. Please indicate how strongly you agree with the statement below. ‘Anaesthetists are uniquely placed to lead the future development of perioperative medicine.’

- Strongly agree
- Agree
- Neither agree nor disagree
- Disagree
- Strongly disagree

26. Who you think should deliver perioperative medicine in your hospital? Please mark all that apply.

- Anaesthetists
- Intensivists
- General physicians
- Organ specific physicians
- Geriatricians
- Other (please specify)

27. Anaesthetists are being asked to increase their input into perioperative services. We have listed some factors that may be seen as barriers to this. Please indicate your views below. (Options for all responses: Strongly agree/ Agree/ Neither agree nor disagree/ Disagree/ Strongly disagree)

- Anaesthetists do not want to practice perioperative medicine
- Increased anaesthetic input into the perioperative pathway will not affect patient outcomes
- There is no room in current job plans
- Anaesthetists lack sufficient training in perioperative medicine
- Physicians are better placed to deliver perioperative medicine
- Surgical teams are reluctant to allow increased anaesthetic input into perioperative services
- The implementation of innovative pathways of care is not supported by the management at my trust
- The cost of increasing perioperative services would be too high
- There are not enough anaesthetists to deliver perioperative medicine
- There is a lack of evidence linking increased perioperative anaesthetic input with improved patient outcomes
- Physicians are encroaching on perioperative medicine

28. Since the RCoA recommendations on perioperative medicine were made, have you instigated any changes within your hospital to help deliver these aspirations?

- Yes
- No
- Not sure

29. If you have instigated change, please use the comment box below to indicate what has been done. Examples may include arranging or attending teaching, business planning, developing liaison pathways etc.
